# Diagnosis in Acute Toothache

**Published:** 1909-05-15

**Authors:** 


### Dental Department.

### DIAGNOSIS IN ACUTE TOOTHACHE.

The diagnosis of odontalgia presents few difficulties. As a rule the patient comes complaining of acute pain in a tooth, and if asked to put a finger-tip on the tooth which he thinks is the cause of his trouble, he is often successful in indicating the exact tooth which is at fault; and almost invariably this tooth is the subject of dental caries. But sometimes the diagnosis is not quite so simple, for the patient may be unable to localise any particular tooth, though, unless more than one tooth is at fault, he can always say definitely which side of the face is affected. Or the patient may localise a tooth which on thorough examination appears quite free from caries; or may refer the pain to the ear, or the eyes, or the angle of the jaw; or, again, may say the pain is all over the head, or that it jumps about, now to one place, now to another.

It is right to examine first and thoroughly the tooth which the patient suspects to be the origin of his toothache, because his belief is so often correct. If no fault can be found in the tooth pointed out, we examine the teeth in the region from which we know the pain is likely to be referred. We know that, with a carious lower third molar, the pain is often referred to teeth in the neighbourhood of the mental foramen, most often the first premolar; that with any carious upper posterior teeth, the pain may be referred to its antagonist in the lower jaw, and *vice-versâ*.

With pain in the eye we examine carefully the upper incisor teeth, especially the canines; with pain in the ear, or at the angle of the jaw, we should suspect the third molars, especially the lower; at the same time remembering that an irritation of any one of the peripheral endings of the fifth nerve may be referred to any other peripheral termination, or along the course of a terminal branch, but that such referred pain is always unilateral. Pain from irritation of a terminal twig of the fifth nerve is never referred to the opposite side of the face.

If no cavity can be found in a tooth after the most careful examination, bearing in mind how easy it is to overlook an interstitial cavity, or one just at or below the gum margin into which the gum has grown, then any filling in the neighbourhood falls under suspicion, and we have very carefully to consider the advisability of its removal to enable us to examine the condition of the pulp or of the pulp canal; whether to remove it or not depends on the condition of the filling. If it shows the slightest suspicion of being leaky, we remove it; the old chart should be searched for, as we have no knowledge of the condition of the pulp underneath, and we know the filling can be replaced. It is only after focussing attention on each single tooth, and making sure of each individual filling, that we can turn from the dental aspects of the case to a consideration of other possible source of trigeminal irritation, or to a diagnosis of functional neuralgia.

Having localised the faulty tooth, we have a further diagnosis to make; to decide whether the pain is due to a simple hyperæmia of the pulp set up by irritation from moderate caries; whether the case is one of an acutely inflamed pulp due to its exposure through caries; or whether it is an infective pulpitis with acute periodontitis, or an alveolar abscess.

If the patient presents a swelled face, the tissues red and œdematous, and the eye partly closed, it is no doubt an alveolar abscess. On looking into the mouth we notice the tongue is furred and the breath foul. The teeth on the affected side are covered with an accumulation of food, from inability to bear a toothbrush near them. We shall find a tooth perhaps perceptibly raised above its fellows, acutely tender to touch; on gently taking it between thumb and finger, it will move quite easily in its socket, and we shall undoubtedly find a cavity, and very likely a fluctuating swelling somewhere near the apex of the root. This swelling is most often on the labial or buccal side of the teeth, but it may be on the lingual side, when there will be a bulging of the hard palate, extending pernaps to the middle line or tracking back towards the soft palate. In the lower jaw an abscess in connection with a posterior molar may form a large swelling at the angle of the jaw, and with it there is often considerable trismus.

But even when the cause of the trouble is a septic root, by no means does it always give so definite a picture. There may be no swelling and no redness; the tongue may not be furred, and the teeth are clean. Then we proceed to inquire into the character of the pain. Pain of a sharp, shooting, stabbing nature, intensified when hot or cold liquids are taken, worse when the patient gets warm in bed at night, and relieved by pressure, as by the patient grinding on it, indicates a live nerve intensely irritated, with a hyperæmic condition of the pulp. The pain is not made worse by a sharp tap, but to probe the inside of the cavity provokes the most acute pain.

On the other hand, pain in toothache of a grumbling, dull, aching character, not affected by changes of temperature, made worse by pressure, indicates a gangrenous pulp with acute periostitis. Here, on tapping the tooth, sharp pain is felt, and probing the cavity causes no pain.

If these symptoms are not definite enough, and we are still unable to arrive at a conclusion as to the condition of the tooth, the debris and decay from the cavity should be removed very cautiously and gently, widening the field of operation by breaking down with a chisel-shaped instrument the unsupported walls of enamel. Provided no more pressure is used than can be avoided, it will be possible to work right down to the pulp, if it be gangrenous, without giving the patient much pain. In the case of an inflamed pulp, on the other hand, the nearer we get down to it, the more painful becomes the operation, and without coming down to the pulp we can assure ourselves of its exact condition.

